# Utilization of dental services and health literacy by older seniors during the COVID-19 pandemic

**DOI:** 10.1186/s12877-022-02758-5

**Published:** 2022-01-31

**Authors:** Sophia Weber, Elena Günther, Sebastian Hahnel, Ina Nitschke, Angelika Rauch

**Affiliations:** 1 Clinic for Prosthetic Dentistry and Dental Materials Science, Leipzig University Medical Center, 04103 Leipzig, Germany; 2 Clinic of General, Special Care and Geriatric Dentistry, University of Zurich, 8032 Zurich, Switzerland

**Keywords:** COVID-19, geriatric dentistry, gerodontology, health literacy, older seniors, utilization

## Abstract

**Background:**

This study aimed to investigate the utilization of dental services by older seniors during the COVID-19 pandemic and to evaluate their ability of finding, understanding, and using information on COVID-19.

**Methods:**

At the end of February 2021, a survey addressing demographic characteristics of the participants, (pain-associated) utilization of dental services, worries regarding a potential COVID-19 infection, the individual use of protective masks, and difficulties regarding the access to information on COVID-19 (by using the modified European Health Literacy Questionnaire [HLS-EU-Q16]) was developed. It was sent to all patients of the Dental Clinic of University of Leipzig who were either 75, 80, or 85 years old (*n* = 1228). Participation was voluntarily and anonymously; questionnaires had to be returned within six weeks, no reminders were sent.

**Results:**

Of the 439 replies (response rate 35.7%), twelve were excluded from data extraction due to disinterest, dementia, or lack of age information. Of the older seniors, 81.5% (*n* = 348) had utilized at least one dental examination and 54.2% of the dentulous patients (*n* = 199) had attended at least one dental hygiene appointment within the past year. Up to 55.8% of all participants said it was “difficult” or “very difficult” to find, understand, and use information on COVID-19, especially when judging reliability of information presented in the media, which was especially true for seniors with assigned care levels presenting odds ratios up to 5.30.

**Conclusions:**

The investigation revealed a frequent utilization of dental services by older seniors during the COVID-19 pandemic. However, the older seniors encountered difficulties finding, using, and understanding information about COVID-19.

## Background

The overall health of a human being, quality of life, and individual well-being are influenced by oral health [1]. In order to regularly examine the oral cavity and prevent or identify potential diseases, an annual dental examination should be considered obligatory. Krause et al. recently investigated the 12-month prevalence of the utilization of dental examinations in the adult German population [2]. Approximately 75% of the interviewed persons had utilized a dental examination within the past year (investigation period 2009–2012). The lowest utilization was identified in the group of older seniors (> 75 years). Similar results were published in a dental report issued by a German health insurance, which identified a continuously decreasing utilization of dental examinations in seniors older than 80 years with increasing age [3]. With age, planned and preventive dental treatments and utilization of periodic dental services are steadily transforming to a complaint-associated utilization of dental examinations, which is due to increased frailty and multimorbidity of older patients [4]. An investigation by the Robert Koch Institute revealed that approximately one fourth of men and one third of women aged 75 years and older suffer from five or more chronical diseases [5]. Moreover, the number of Germans receiving nursing care is constantly increasing, with approximately 3.4 million people in 2017 [6]. A structured assessment is used to assign a care level (CL) by determining the grade of impairment and the resulting level of needed support, with higher levels representing an increased impairment (CL1 – CL5, for explanations see appendix) [7]. In 2014, the Fifth German Oral Health study identified a correlation between nursing care and the utilization of dental examinations by older seniors (75–100 years). While 62% of older seniors without nursing care used dental examinations on a regular and preventive basis, most same-aged seniors with assigned care levels only attended dental examinations when having complaints although they had an even higher prevalence of carious lesions or losing teeth [8].

In the course of the COVID-19 pandemic dentists are constantly confronted with patients’ concerns regarding a potential infection with the coronavirus through aerosols during dental procedures [9–11]. Especially people older than 50 to 60 years are characterized as high-risk patients for COVID-19 disease [12]. In general, the risk increases with higher age and presence of co-morbidities, such as diabetes mellitus, cardiovascular diseases, or diseases of the respiratory system. An investigation confirmed that patients with recently diagnosed cancer or lung and heart diseases, respectively, have a higher risk to be hospitalized for COVID-19 [13]. If people present both high age and co-morbidities, they are even more at risk [12]. A recent investigation addressed the impact of the COVID-19 pandemic on the utilization of emergency dental services and revealed that after the outbreak of the COVID-19 pandemic, the percentage of people using emergency dental services dropped by 38% [14].

The excessive amount and vast change of information on the coronavirus might have confused and worried patients. Referred to as an “infodemic” (information epidemic) [15], the overload of both reliable and misguiding information rapidly spread through society, which made the need for distinction between reliable and misinformation even more important. Essential skills that are needed to find, understand, and use information that help to foster and maintain a good health status are summarized as “health literacy” [16]. Abel and McQueen stated that health literacy is also about reflecting on one’s own actions in a public health emergency [17], pointing out the importance of a sufficient health literacy during the COVID-19 pandemic.

The current study aimed to investigate the utilization of dental services by older seniors in the urban area of Leipzig, Germany, during the COVID-19 pandemic. Moreover, the health literacy of patients during the pandemic should be evaluated. The null hypotheses suggested a similar utilization of dental services and similar health literacy of older seniors with and without an assigned care level.

## Methods

### Survey

A questionnaire that consisted of three parts was developed. The first part of the questionnaire gathered data regarding demographic characteristics of participants such as age, sex, allocated care level, and the COVID-19-vaccination status. The second part addressed patients’ utilization frequency of dental examinations and dental hygiene appointments from the beginning of March 2020 until the end of February 2021 as well as their pain-associated utilization of such. Moreover, participants should describe if they were worried about an infection with COVID-19 when being with friends and family, at a dental practice, or in everyday life. They were asked to indicate whether they usually wear a mask and to specify the type of mask. The last section of the survey included a modified version of the European Health Literacy Questionnaire (HLS-EU-Q16 [18]) reduced to 10 questions.

For recruitment of participants, an information sheet, the guidelines of data protection, the questionnaire, and a stamped and addressed return envelope were sent to all patients of the Dental Clinic of the University of Leipzig who were 75, 80 or 85 years old and who had attended an appointment since 10/2019 – this date is due to a general new setup of the patient database in the Dental Clinic of Leipzig University (*n* = 1228; Table 1). All questionnaires were dispatched at the end of February 2021. Participants were invited to complete the questionnaire and return it anonymously by the end of March 2021. Due to the fact that participation was voluntarily, consent to participate was given by returning the survey. All responses until April 14th, 2021 were taken into consideration; no reminders were sent. The study was conducted according to the guidelines of the Declaration of Helsinki. Concepts and questionnaires of the survey were reviewed and approved by the ethical committee of Leipzig University (005/21-ek).

**Table 1:** Age and sex distribution of patients receiving the survey

	Patients receiving the survey		
Age group [years]	Total % (n)	Male % (n)	Female % (n)
75	26.9 (331)	13.0 (160)	13.9 (171)
80	43.2 (530)	21.0 (258)	22.2 (272)
85	29.9 (367)	14.1 (173)	15.8 (194)
Overall	100 (1228)	48.1 (591)	51.9 (637)

### Pretesting of the survey

In December 2020, pretesting of the questionnaires was performed to verify practicability and identify potential problems using the think-aloud strategy. Thirteen participants aged 70–87 years (mean age 81 years, 62% female) without previous dental treatment at Leipzig University were recruited. Based on the pretesting results, some questions of the first and second part of the survey were rephrased for easier comprehension. In addition, synonyms were added to terms that were difficult for the test participants to understand.

### Statistical analysis

Due to possible birthdays during the survey phase, data of participants belonging to the requested age group ±1 year were included. Statistics were performed determining frequencies and their 95% confidence intervals (CI) with bootstrap (IBM SPSS 27, IBM, Armonk, NY, USA). Differences of dental service utilization and answers to questions of the modified HLS-EU-Q16 were assessed in dependence on the assigned care level (CL) using Chi-square tests. Odds ratios (OR) and their 95% CI were calculated in this context. The level of significance was set to p < 0.050.

## Results

### Demographic data of participants

**Fig. 1:**
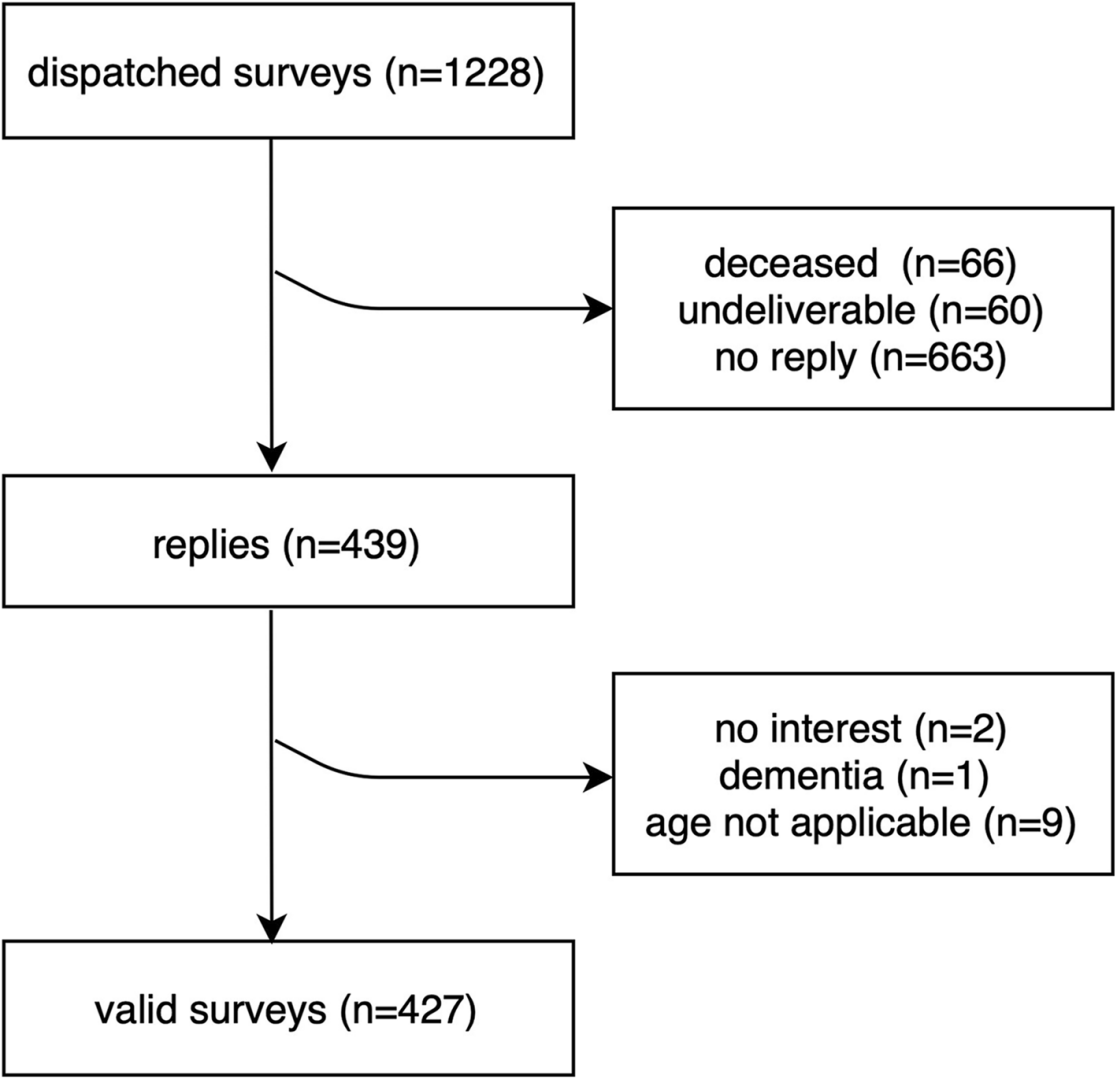
Overview of responses; information on deceased participants were conveyed by relatives

A total of 439 replies arrived at the Department of Prosthodontics and Dental Materials Science at Leipzig University (Fig. 1), representing a response rate of 35.7%. After extracting invalid questionnaires, 427 valid replies (34.8%) remained.

Approximately one fourth of the 427 participants belonged to the age group of 75 years (27.9%, *n* = 119), almost half of the participants to the age group of 80 years (46.8%, *n* = 200), and another fourth to the age group of 85 years (25.3%, *n* = 108). Of the participants, 50.4% were female (Table 2).

**Table 2:** Age and sex distribution of participants

	Participants		
Age group [years]	Total % (n)	Male % (n)	Female % (n)
75	27.9 (119)	48.7 (58)	51.3 (61)
80	46.8 (200)	48.0 (96)	52.0 (104)
85	25.3 (108)	53.7 (58)	46.3 (50)
Overall	100 (427)	49.6 (212)	50.4 (215)

Most of the participants (77.8%; *n* = 332) did not have an assigned care level (CL_no_) and four participants did not report it. Therefore, 21.3% (*n* = 91) of the respondents officially needed professional medical care (CL_yes_: CL1 4.2%, *n* = 18; CL2 10.3%, *n* = 44; CL3 6.3%, *n* = 27; CL4 0.2%, *n* = 1; CL5 0.2%, *n* = 1). Concerning the COVID-19-vaccination status, about one third (32.1%, *n* = 137) had received at least one injection of vaccine, whereas almost two thirds (65.1%, *n* = 278) had not been vaccinated yet; the remaining participants (2.8%, *n* = 12) did not report their vaccination status.

### Utilization of dental services

**Fig. 2:**
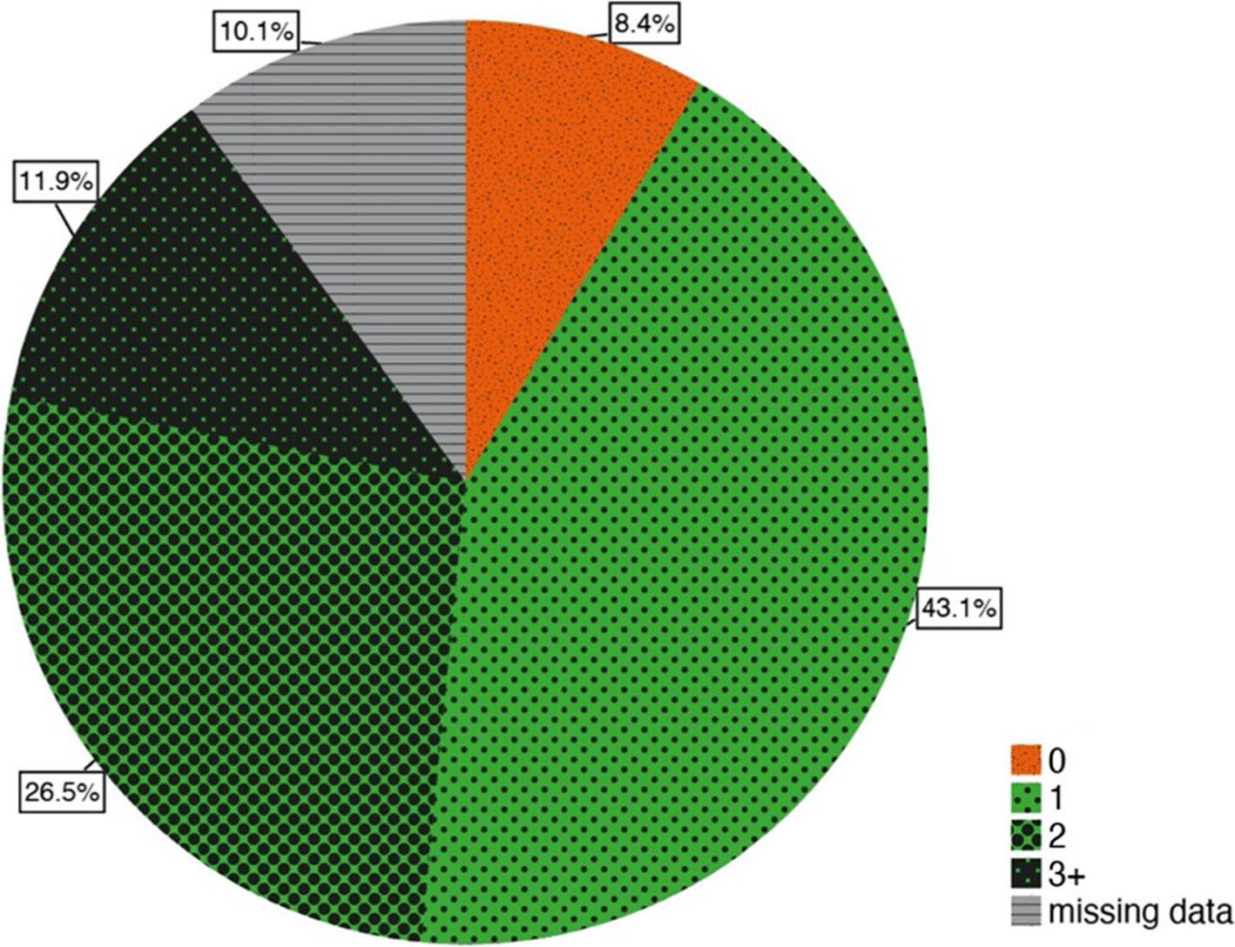
Frequency of dental examinations, *n* = 427

The majority of the participants had utilized at least one dental examination within the last year (81.5%, *n* = 348; Fig. 2), whereas the remaining participants did either not utilize a dental examination (8.4%, *n* = 36) or did not report about it (10.1%, *n* = 43). There were no statistically significant differences between the utilization of at least one dental examination of seniors without or with assigned care levels (CL_no_/CL_yes_ 91.2/88.5%; *N* = 384). Eleven participants (2.6%) answered that they did not meet a dentist even though they were having pain.

**Fig. 3:**
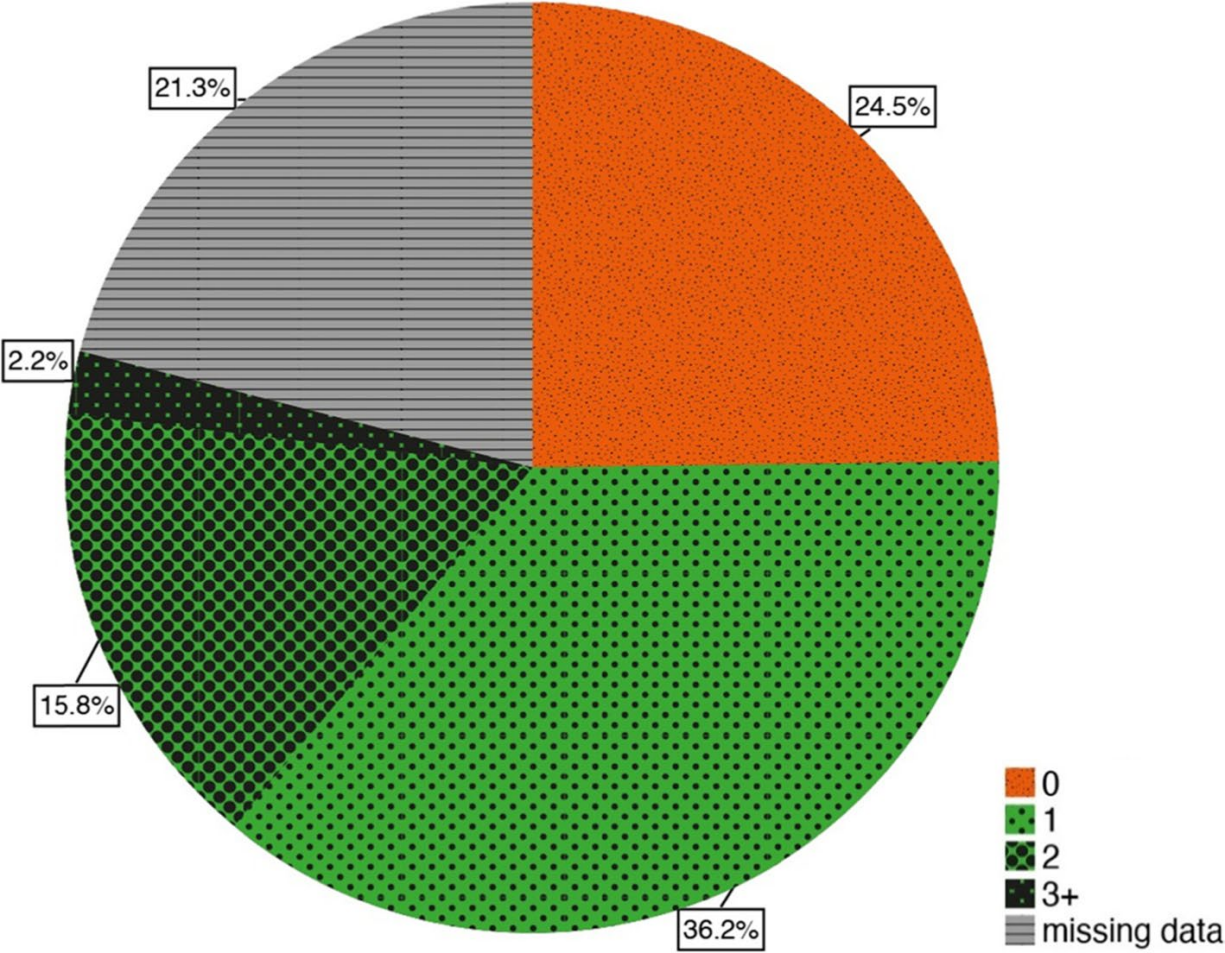
Frequency of dental hygiene appointments, *n* = 367, edentulous patients (*n* = 60) excluded

Sixty of the participants stated that they were edentulous; thus, in terms of dental hygiene appointments, they were not included in data analyses. Almost half of the dentate older patients had used at least one dental hygiene appointment (54.2%, *n* = 199, Fig. 3). The other half of the patients had either not used the service (24.5%, *n* = 90) or did not report the use of such (21.3%, *n* = 78). There was no statistically significant difference for the utilization of at least one dental hygiene appointment in dependence on the existence of an assigned care level (CL_no_/CL_yes_ 70.9/59.6%; *N* = 289).

Regarding the spread of the coronavirus, 37.8% (*n* = 159, *N* = 421) were concerned about transmission during daily activities such as grocery shopping or going to the gym/hairdresser, 21.8% (*n* = 91, *N* = 417) feared contagion when spending time with the family, and 21.3% (*n* = 89, *N* = 418) when being at a dental practice/clinic.

Concerning the individual use of facemasks, 3.3% (*n* = 14, *N* = 426) stated that they were not using masks, 1.6% (*n* = 7, *N* = 426) wore self-sewn masks, 17.4% (*n* = 74, *N* = 426) used surgical masks, and 77.7% (*n* = 331, *N* = 426) used particle filtering masks, e. g., FFP2 masks.

### Health literacy during the COVID-19 pandemic

The answers to the modified European Health Literacy Questionnaire revealed that 13.5% (*n* = 55) to 55.8% (*n* = 227) of the participants perceived difficulties (“difficult” or “very difficult”) regarding health literacy (Table 3). The topic that was rated (very) difficult by most patients addressed the question, whether it was difficult to judge if the information on COVID-19 in the media is reliable (55.8%), followed by how to behave in case of a COVID-19 infection (41.8%) and where to get professional help (40.2%).

**Table 3:** Modified European Health Literacy Questionnaire (HLS-EU-Q16) and assigned health domain (HC: Health Care; DP: Disease Prevention; HP: Health Promotion). Frequency and odds ratios (OR) of answers “difficult” and “very difficult” in percent (%); absence/presence of care level (CL_no_/CL_yes_); 95% confidence interval (CI)

Item Number HLS-EU-Q16		Question	Health domain	Answers “difficult” and “very difficult” % [95% CI]	OR [95% CI]
Modi-fied	Origi-nal				
		How easy would you say it is to...			
1	1	… find information on COVID-19?	HC	13.5 [9.5–16.7]	2.83 [1.54–5.22]
2	13	… find out about activities that are good for your mental well-being during COVID-19 pandemic (go for a walk, Pilates, etc.)?	HP	21.0 [17.5–26.1]	5.30 [3.11–9.05]
3	8	… find information on how to manage mental health problems (Depression, anxiety, etc.) during COVID-19 pandemic?	DP	27.5 [22.8–32.2]	1.58 [0.94–2.65]
4	9	… understand health recommendations/ behaviors for your protection against COVID-19?	DP	16.6 [12.6–20.3]	2.95 [1.67–5.19]
5	3	… understand a potential treatment of COVID-19?	HC	28.9 [23.3–32.8]	2.27 [1.37–3.76]
6	16	… judge if/which everyday behavior increases your risk of a COVID-19 infection?	HP	27.8 [23.6–33.1]	1.90 [1.15–3.16]
7	12	… decide how you can protect yourself from COVID-19 based on information in the media?	DP	22.5 [18.0–26.8]	1.44 [0.83–2.47]
8	11	… judge if the information on COVID-19 in the media is reliable?	DP	55.8 [49.7–60.2]	1.25 [0.77–2.03]
9	2	… find out where to get professional help when infected with COVID-19?	HC	40.2 [34.8–45.1]	1.79 [1.10–2.89]
10	6	… decide on how to behave in case of a COVID-19 infection with the available information?	HP	41.8 [35.3–46.2]	1.70 [1.05–2.74]

When analyzing answers in dependence on the participants’ assigned care level, statistically significant differences were identified for seven of the ten questions. Highest ORs were observed for perceiving it as difficult

- to find out about activities that are good for mental well-being (CL_no_/CL_yes_ 13.8/45.9%; OR 5.30 [3.11–9.05]),
- to understand health recommendations/behaviors (CL_no_/CL_yes_ 13.0/30.6%; OR 2.95 [1.67–5.19]), and
- to find information on COVID-19 (CL_no_/CL_yes_ 10.4/24.7%; OR 2.83 [1.54–5.22]).

## Discussion

According to the results of the current investigation, there was a high utilization of dental examinations (81.5%) and dental hygiene appointments (54.2%) by older seniors despite the COVID-19 pandemic, which was independent on an assigned care level. However, the older seniors encountered difficulties in finding, understanding, and using information regarding COVID-19, especially when they had an assigned care level. Thus, the null hypotheses of this investigation were partially rejected.

The high response rate to the questionnaire indicated a high activity level of the older seniors in Leipzig. Of the respondents, 21.3% stated that they had an assigned care level, which corresponds to nationwide data from Germany [19]. Regarding the vaccination status, the rate of the respondents with at least one vaccination shot (32.1%) was higher than the average vaccination rate in Germany (26.2%) [20] and Saxony (19.9%) [21]. This might be because older seniors are categorized as highest priority for vaccination in Germany [22], whereas national and federal data include all age and priority groups.

The analysis of the older patients’ utilization of dental services represents a higher utilization rate than reported by the population-representative studies “Health in Germany up-to-date” (GEDA; 75.3%) [2]. Considering that several studies revealed a decrease in utilization of dental services due to the COVID-19 pandemic [23–25], a frequency of 81.5% observed in the present study is high. This might be due to the fact, that dental clinics are considered institutions with a high standard of hygiene, implicating a self-perceived low risk for a virus transmission [26]. Another reason for a high utilization rate might be the fact that participants of the investigation were patients of the Dental Clinic of Leipzig University, that promotes a consistent recall system. There are personal reminders regarding annual examinations or dental hygiene appointments, which might have helped reducing fears or worries toward a possible COVID-19 infection during dental treatment. Moreover, the Medical Clinic of the University of Leipzig has a good reputation among the population, especially as COVID-19 patients from other countries were successfully treated in both the medical and dental clinic. This phenomenon may have contributed to the high utilization of dental services. Moreover, as part of the German statutory health care system, patients attending at least one dental check-up per year qualify for monetary boni, which might have been a strong external motivation to attend a dental check-up. However, no information on the date of the dental visit was assessed in the current study; thus, older seniors could have utilized the dental services in the summer of 2020, when low incidence rates of the coronavirus were recorded in Germany.

Nonetheless, the utilization of dental hygiene appointments was lower than the utilization of dental examinations. This phenomenon might be explained by the fact that, in Germany, costs for dental examinations are fully covered by statutory health insurances, while professional cleaning of teeth and dentures usually requires additional payment by the patients themselves.

One third of the respondents primarily feared daily activities such as grocery shopping or going to the gym/hairdresser regarding a potential transmission of the coronavirus. This is not surprising as particularly indoor activities are supposed to facilitate COVID-19 infections [27, 28] and physical activities or speaking loudly seem to increase the risk of spreading the coronavirus [29]. Although these risk factors also apply to spending time with family and friends or going to a dental practice, only a fifth of the participants considered these issues as high-risk activities. Older seniors might tend to trust family and friends more than foreigners, even though they usually allow more intense physical contact or increased time together. Regarding dental practices, the high hygiene standards that already existed pre-pandemic accompanied by the rapid advancement of COVID-19-specific hygiene concepts might have supported faith in the dental/medical institutions. This assumption is supported by data of the German Dental Association, revealing that 88% of Germans consider dental practices as institutions with a high standard of hygiene [26].

Analysis of health literacy responses revealed that more than half of the participants found it difficult to judge the reliability of information on COVID-19 purported in the media, which emphasizes the assumption of an “infodemic” during the Corona crisis. Digital media are important for acquiring knowledge quickly, and a high level of digital literacy is required to find, evaluate, and use information. Limited access to digital media among older seniors might reduce possibilities to retrieve information, while lower digital skills can complicate a differentiation between information of trustworthy sources and dishonest ones, e. g., disinformation campaigns [30]. In addition, latest information on COVID-19 transmission rates, behavior in case of an infection or information on professional help (e. g., corona tests or vaccination appointments) were usually published online first. These issues might have caused about 40% of the participants to state that they found it difficult to judge how to behave in case of an infection and where to get professional help. Besides, the survey was conducted during the “third wave” of the COVID-19 pandemic, which was characterized by an overload of information addressing, e. g., vaccination, risk areas, or masks. Moreover, there was a high level of frustration since restrictions in everyday life had been ongoing for several months. According to results of the modified HLS-EU-Q16, difficulties were increased when participants had an assigned level of care. Considering that over 70% of all participants requiring care were assigned to at least CL2 – representing a major impairment or even more – it seems a logical consequence that these people rate it increasingly difficult to find, understand, and use latest information.

The limitations of the current study include that it was not possible to assess, whether the utilization rates of dental services increased or decreased in the same cohort in comparison to the situation before the pandemic. Furthermore, a recall bias is likely. As questions of the HLS-EU-Q16 had been modified it was not possible to determine a sum score comparable to reference values; however, frequencies determined for each question can be used for future comparisons. Moreover, the evaluated data only apply to the group of patients who are treated at the Dental Clinic of the University of Leipzig. As a clinic is usually operating differently than a common German dental practice with one or few dentists, the results of the present investigation do not represent the entire older German population. In order to gain a more comprehensive overview, a survey among same-aged patients of dental practices should be performed.

## Conclusion

According to the results of this investigation, there was a high utilization rate of dental services despite the COVID-19 pandemic. Older seniors felt it was difficult to find, use and understand information on COVID-19 purported in the media, especially when judging the reliability of these information. Older people with an assigned care level were affected from difficulties in terms of health literacy more frequently. In times of increasing digitalization, it seems important to supply older and frail seniors with trustworthy and current information on important health issues by using reliable and preferably non-digital sources. The burdens arising from the COVID-19 pandemic highlight that dental recall systems should be adjusted to the individual needs of all patient groups.

## Data Availability

The datasets used and analyzed during the current study are available from the corresponding author on reasonable request.

## Appendix

### Care levels

In Germany, five care levels are distinguished. The degree of care is based on how independent a person acts and what abilities he or she still has. The classification is based on the following six categories: mobility, cognitive and communicative abilities, behavioral and psychological problems, self-care, independent handling of illness- or therapy-related requirements, and organization of everyday life and social contact. During the examination, the evaluator assesses up to 16 criteria per category and assigns scores. The higher the score the higher the care level and consequently the required support (CL1: sum score 12.5 ≤ 27, CL2: sum score 27 ≤ 47.5, CL3: sum score 47.5 ≤ 70, CL4: sum score 70 ≤ 90, CL5: sum score 90–100). Whereas care level 1 represents the least impairment of independence or abilities of a person, care degree 5 corresponds to the most severe limitations.

## Abbreviations

CI: Confidence interval
CL: Care level
e. g.: Exempli gratia; for example
Fig.: Figure
HLS-EU-Q16: European Health Literacy Survey
n: Count
N: Total participants responding to the question
OR: Odds ratio
